# Mania: An atypical presentation of probable *Streptococcus agalactiae* meningoencephalitis

**DOI:** 10.1016/j.idcr.2023.e01817

**Published:** 2023-06-09

**Authors:** Ann Hudson, Daniel Bobo, Libardo Rueda Prada, Igor Dumic, Emilia Petcu, Milena Cardozo, Fnu Shweta

**Affiliations:** a Mayo Clinic Alix School of Medicine, Rochester, MN, USA; b Hospital Medicine, Mayo Clinic Health System, Eau Claire, WI, USA; c Department of Family Medicine, Mayo Clinic Health System, Eau Claire, WI, USA; d Hospital Medicine, Mayo Clinic, Jacksonville, FL, USA; e Department of Infectious Diseases, Mayo Clinic Health System, Eau Claire, WI, USA

**Keywords:** Streptococcus agalactiae, Meningitis, Encephalitis, Mania, Delirium

## Abstract

*Streptococcus agalactiae*, also known as Group B Streptococcus (GBS), is a common pathogen in the neonatal period, causing meningitis and sepsis. In non-pregnant adults it is an unusual cause of meningitis. We report about an elderly female with several risk factors for invasive GBS infection who developed GBS meningoencephalitis one month after treatment for COVID-19 upper respiratory tract infection. The patient presented with mania, and the classic triad of headache, neck stiffness, and fever was absent which contributed to the delay in diagnosis. Following initiation of treatment with intravenous ceftriaxone she attained full recovery, and her behavior returned to baseline. This case illustrates an unusual presentation of an emerging infection and should alert clinicians about this presentation. By reporting this case we want to raise awareness about mania as a presenting feature of meningoencephalitis. This should lead to more timely diagnosis and better outcomes for future patients.

## Introduction

Acute bacterial meningitis is a medical emergency. It has a classical presentation of fever, headache, and nuchal rigidity that can make this diagnosis relatively easy to establish. However, when these typical symptoms and clinical findings are missing, establishing diagnosis is more challenging especially if presenting with unclear or mixed neuropsychiatric symptoms [1], [2], [3]. Meningitis can develop with concurrent inflammation of the brain parenchyma (encephalitis) in which case it is termed meningoencephalitis [2]. In such cases, more atypical and insidious presentations occur, and patient will experience hallucinations, agitation, personality/behavioral changes, altered sensorium, or psychosis that can result in diagnostic difficulties and delay in appropriate treatment [2].

The most common causes of community acquired meningitis in USA adults remain *Streptococcus pneumoniae*, *Neisseria meningitidis, Listeria monocytogenes* and *Haemophilus influenzae* [3]. In up to 50% of patients with acute encephalitis the cause remains unknown, while among identified pathogens, herpes simplex virus (HSV) is the most common cause [4]. Encephalitis due to GBS has not been reported previously.

*Streptococcus agalactiae*, commonly known as group B streptococcus (GBS) is the most common bacterial cause of meningitis in infants within the first 3 months of life [5]. It is an exceedingly rare cause of meningoencephalitis in adults [6]. Infection induced mania is more commonly described in viral encephalitis [7]. Mania has been very rarely described as presenting manifestation of bacterial meningitis [8] and has not been reported with GBS meningitis thus far.

## Case presentation

A 74-year-old woman was admitted for dry cough, chest pain, and shortness of breath. One month prior she was hospitalized and treated for COVID-19 upper respiratory tract infection, and she recovered successfully with treatment of monoclonal antibodies.

Her pertinent medical and psychiatric comorbidities included hypertension, hyperlipidemia, diabetes mellitus, obesity, metabolic syndrome associated fatty liver disease (MAFLD), generalized anxiety disorder, and splenectomy following a remote motor vehicle accident. She was a former smoker, consumed 5 alcoholic beverages per week and did not report any illicit drug abuse. She was married with adult children. Her medication included mirtazapine 7.5 mg at bedtime and paroxetine 20 mg daily for anxiety. Her post-splenectomy vaccinations were up to date.

On presentation she was noted to be afebrile with a temperature of 36.6 °C, heart rate 88 beats per minute, respiration 18 per minute, blood pressure 107/90 mmHg, and an oxygen saturation of 86% on room air which improved to 99% on 2 L of supplemental oxygen. Physical examination revealed a pleasant, alert, talkative female who was oriented to person, place, and time. She was not in acute distress. There was no evidence of head trauma, pallor, or nystagmus. Her lung examination was positive for rales bilaterally. Neurological examination revealed no nuchal rigidity, or focal neurological deficits. The remainder of her physical exam was normal.

Initial laboratory evaluation showed a white blood cell (WBC) of 11 800/μL with neutrophilia. Electrolytes, renal and liver function tests were within normal range. Glucose was 170 mg/dl, procalcitonin 0.19 ng/ml, and C reactive protein (CRP) was 13.3 mg/dL ( normal less than 5 md/dL). Blood cultures showed no growth after 5 days of incubation. Sputum cultures could not be obtained. CT chest angiogram showed no pulmonary embolism but chronic appearing interstitial changes with ground-glass opacities in the bases suspicious for infectious pneumonitis. Ultrasound of the lower extremity showed no deep venous thrombosis. Transthoracic echocardiogram showed ejection fraction of 60% and concentric remodeling of the left ventricle without regional wall motion abnormalities. There were no valve vegetations. Based on her symptoms of cough, presence of leukocytosis and findings of infiltrates on chest CT the patient was started on cefepime and doxycycline for possible pneumonia.

The following day, the patient started exhibiting paranoid psychotic behavior with suspicion of visitors and staff. The family reported no history of similar symptoms and unanimously vouched that she has been a caring, loving wife, and parent. Repeat CT chest showed improving infiltrates. Mirtazapine dose was increased to 15 mg at bedtime, and buspirone 15 mg twice daily was added. Symptoms were initially attributed to delirium in the setting of acute illness. However, this was later ruled out due to absence of cognitive dysfunction, lack of attention deficit, without fluctuation in consciousness.

The patient’s psychiatric symptoms worsened, and she started manifesting manic symptoms of pressured speech, flight of ideas, racing thoughts, expressions of grandiosity. She began to demand family to purchase things that she needed and ordered a variety of candy, offering gifts to staff. She was constantly requesting to go to the gift shop and had developed a spending spree. She was noted at times to wear heavy makeup, dark lipstick, fake lashes, and a bright flowered hat. Due to the patient's mania, she was started on lithium 300 mg twice daily while mirtazapine and paroxetine were discontinued. On hospital day 3 the antimicrobials were changed from cefepime and doxycycline to levofloxacin.

Her WBC continued to rise to18 800/μL, although she remained afebrile. The patient initially denied symptoms of headache and neck stiffness, however eventually these were reported. CT head without IV contrast showed no acute pathology. Ammonia level (29 mmol/L), pCO2 (38 mg Hg), and lactate (2.2 mmol/L) were normal. Autoimmune workup including testing for NMDA encephalitis was negative. A lumbar puncture was performed, and cerebrospinal fluid (CSF) analysis showed clear fluid with zero total nucleated cells, zero erythrocytes, protein was 30 mg/dl, and glucose 89 mg/dl. CSF cultures were negative, but polymerase chai reaction (PCR) was reported positive for *Streptococcus agalactiae.*

The patient was diagnosed with *Streptococcus agalactiae* meningoencephalitis. She was treated with ceftriaxone 2 g every 12 h for a total course of 2 weeks. Following initiation of ceftriaxone, her manic symptoms resolved, lithium was stopped, and her behavior returned to baseline. She was transferred to a post-acute care unit for completion of intravenous antibiotics. Ultimately the patient was discharged home with significant improvement in clinical and mental status and she remains off lithium and with normal behavior.

## Discussion

GBS is one of the leading causes of meningitis in neonates but remains rare in adults accounting for approximately 1.3% of all community-acquired bacterial meningitis in this group [9]. Older patients (above age of 65), and those with comorbidities including diabetes mellitus, liver disease, and immunosuppression have been found to be at a higher risk of acquiring invasive GBS infections and dying from it [10]. However, cases of GBS meningitis have been reported even in previously healthy adults with no risk factors [11], [12], [13], [14]. In some patients, yet undiagnosed genetic abnormalities, might predispose for development of meningitis as seen in a case report of a young healthy adult who developed GBS, and whose family history was significant for recurrent episodes of bacterial meningitis in multiple family members [13].

Our patient had several risk factors such as obesity, diabetes mellitus, splenectomy, and MAFLD which has been recently found to be risk factors for development of various infections, including pneumonia [15].

GBS is a Gram-positive, encapsulated pathogen that causes beta hemolysis on blood agar. It is a catalase negative streptococcus, and facultative anaerobe that colonizes human skin, respiratory, gastrointestinal, and urogenital tract [16], [17]. The exact incidence of invasive GBS in adults is not clear, but an increase in the last decade has been documented. In the United States, GBS infections increased from 8.1 cases /100 000 in 2008–10.9 cases/100 000 in 2016 [18]. A study from France found that capsular type III strain was overrepresented in cases of meningitis [19]. A study from Thailand, of non-pregnant adult patients demonstrated similar results that primary bacteremia was the most common manifestations of GBS invasive disease, followed by arthritis, cellulitis, osteomyelitis, endocarditis, and meningitis [20]. In this study mortality was 17% and predictors of mortality in univariate analysis were found to be: age above 65 years, altered mental status, absence of fever, shock, and high Pitt bacteremia score (above 4).

Our patient was partially treated with broad spectrum antimicrobials including cefepime, doxycycline, and levofloxacin which might have contributed to the absence of typical CSF findings in bacterial meningitis. However, isolation of *Streptococcus agalactiae* from CSF by PCR (BioFire, Film Array- meningitis/encephalitis panel kit, Mayo Medical Laboratories) supports the diagnosis of GBS meningoencephalitis in patient on broad spectrum antibiotic therapy. Prior antibiotic therapy could also explain the lack of relatively normal CSF studies. CSF is sterile and isolation of GBS from it should not be considered colonization. Adults previously treated with antibiotics as short as one day duration may have negative GBS culture from CSF [21]. Prolonged GBS CSF PCR positivity has been demonstrated to be related to persistent infection in a neonate with ventriculitis and residual brain abscess [22] suggesting that test positivity can be associated with residual infection. An Irish multicenter diagnostic accuracy study comparing CSF PCR with culture in infants with late-onset GBS meningitis found the test to be 100% sensitive with a specificity of 97% [23]. Cases were reviewed to confirm true or probable infection. Another single center study found that CSF PCR had a sensitivity of 60% and specificity of 97% for GBS invasive disease [24]. No similar studies have been found in the adult patient population.

Molecular detection assays increase diagnostic accuracy for bacterial infections in individuals who are receiving or have received broad spectrum antimicrobials that may decrease sensitivity of bacterial cultures [25], [26], [27], [28]. Bacterial maningitis with normal CSF analysis has been previously reported [29].

It was reported that approximately 10% of all patients with invasive GBS have central nervous system (CNS) involvement [30]. Neurological complications of invasive GBS CNS disease include epidural abscess, acute bacterial meningitis, and meningo- ventriculitis [30]. Classically bacterial meningitis is characterized by acute onset of fever, headache, and neck stiffness. The presence of all 3 of these symptoms simultaneously is present in less than 50% of cases [31]. Other clinical manifestations include cranial nerve (CN) palsies because of increased intracranial pressure, especially CN III, IV, VI, and VII, and focal neurologic signs, because of brain ischemia [32]. Cerebral infarction as initial presentation of GBS meningitis has been reported [33]. The source of infection is not always found, and one study identified the source of infection in only one third of patients [9]. Similarly, while we did not find the source of infection in our patient it was preceded by pneumonia and COVID-19 infection which might have damaged respiratory epithelium and lead to CNS infection and meningitis.

This case is an example of a similar conundrum in initial diagnosis and treatment with delirious mania like symptoms but due to bacterial meningoencephalitis.

Complications are common in patients with GBS meningitis. These patients frequently have bacteremia with or without endocarditis and seeding of infection to distant organs. Cases of septic arthritis, necrotizing fasciitis, discitis, osteomyelitis, and endophthalmitis have been reported [9], [34], [35]. Of these endophthalmitis is of particular importance as patients frequently do not recover vision. Additionally, if timely recognized, intravitreal antibiotic administration or vitrectomy might be carried out which might improve the outcome in this subgroup of patients with invasive GBS. In the case of isolated GBS meningitis therapy usually consists of intravenous cephalosporin (typically 3rd generation) given over a two-week period. If there are complications such as endocarditis, osteomyelitis, endophthalmitis, and/or septic arthritis, the duration of antimicrobials might be longer.

GBS was considered to be universally susceptible to penicillin and cephalosporins. However, penicillin resistant GBS (PRGBS) have been reported in Japan, United States, and Canada [36], [37]. The rates of PRGBS isolates seem to be increasing so this is something that must be kept in mind when choosing appropriate antimicrobial therapy. Since there were no culture results available for our patient to determine sensitivity to penicillin, she was treated with ceftriaxone to complete course of therapy. Corticosteroid therapy has been shown to improve morbidity and mortality in patients with *Streptococcus pneumoniae* meningitis, and in children with *Hemophilus influenzae* meningitis, but such benefit was never documented for meningitis due to GBS.

## Conclusion

We report about an elderly female with multiple risk factors for invasive GBS, including advanced age, MAFLD, diabetes, and splenectomy. The patient presented unusually with signs and symptoms of mania and due to non-specific presentation, the diagnosis was somewhat delayed. By reporting this case, we want to highlight this unusual manifestation of an emerging infection and point towards specific risk factors for GBS meningitis development.

## Consent

None.

## Ethical approval

Na.

## Declaration of Competing Interest

Authors declare no conflicts of interest and agree to pay APC charges.

Dr Shweta FNU is Associate Editor for the journal ID cases. Other co-authors do not have any conflicts of interest.
